# Pan-Cancer Study on Protein Kinase C Family as a Potential Biomarker for the Tumors Immune Landscape and the Response to Immunotherapy

**DOI:** 10.3389/fcell.2021.798319

**Published:** 2022-01-31

**Authors:** Alaa Abdelatty, Qi Sun, Junhong Hu, Fubing Wu, Guanqun Wei, Haojun Xu, Guoren Zhou, Xiaoming Wang, Hongping Xia, Linhua Lan

**Affiliations:** ^1^ Department of Pathology in the School of Basic Medical Sciences and Key Laboratory of Antibody Technique of National Health Commission, Nanjing Medical University, Nanjing, China; ^2^ Department of Pathology, Faculty of Veterinary Medicine, Kafrelsheikh University, Kafrelsheikh, Egypt; ^3^ Department of Pathology, Nanjing Drum Tower Hospital, The Affiliated Hospital of Nanjing University Medical School, Nanjing, China; ^4^ Department of Colorectal and Anal Surgery, The First Affiliated Hospital of Zhengzhou University, Zhengzhou, China; ^5^ Sir Run Run Hospital, Nanjing Medical University, Nanjing, China; ^6^ Department of Oncology, Jiangsu Cancer Hospital and The Affiliated Cancer Hospital of Nanjing Medical University and Jiangsu Institute of Cancer Research, Nanjing, China; ^7^ Department of Hepatobiliary Surgery, The First Affiliated Hospital of Wannan Medical College (Yijishan Hospital of Wannan Medical College), Wuhu, China; ^8^ Key Laboratory of Diagnosis and Treatment of Severe Hepato-Pancreatic Diseases of Zhejiang Province, The First Affiliated Hospital of Wenzhou Medical University, Wenzhou, China

**Keywords:** protein kinase C, pan-cancer study, biomarker, tumors immune landscape, immunotherapy

## Abstract

The protein kinase C (PKC) family has been described with its role in some cancers, either as a promoter or suppressor. PKC signaling also regulates a molecular switch between transactivation and transrepression activity of the peroxisome proliferator-activated receptor alpha (PPARalpha). However, the role of different PKC enzymes in tumor immunity remains poorly defined. This study aims to investigate the correlation between PKC genes and tumor immunity, in addition to studying the probability of their use as predictive biomarkers for tumor immunity and immunotherapeutic response. The ssGSEA and the ESTIMATE methods were used to assess 28 tumor-infiltrating lymphocytes (TILs) and the immune component of each cancer, then correlated with PKC levels. Prediction of PKC levels-dependent immunotherapeutic response was based on human leukocytic antigen (HLA) gene enrichment scores and programmed cell death 1 ligand (PD-L1) expression. Univariate and multivariate Cox analysis was performed to evaluate the prognostic role of PKC genes in cancers. Methylation level and CNAs could drive the expression levels of some PKC members, especially PRKCI, whose CNGs are predicted to elevate their level in many cancer types. The most crucial finding in this study was that PKC isoenzymes are robust biomarkers for the tumor immune status, PRKCB, PRKCH, and PRKCQ as stimulators, while PRKCI and PRKCZ as inhibitors in most cancers. Also, PKC family gene levels can be used as predictors for the response to immunotherapies, especially HLAs dependent and PD-L1 blockade-dependent ones. In addition to its prognostic function, all PKC family enzymes are promising tumor immunity biomarkers and can help select suitable immune therapy in different cancers.

## 1 Introduction

Cancer is a multifactorial disorder comprising tumor-immune system interactions (Finn, 2008). Consecutive studies have proved the crucial role of immune components in attacking the nascent transformed malignant cells, based on the tumor immunosurveillance theory (Zhang et al., 2003; Galon et al., 2006; Jochems and Schlom, 2011). In this regard, many developed immunotherapeutic drugs aim to recruit the immune cells to recognize and fight cancers (Harris and Drake, 2013; Shimabukuro-Vornhagen et al., 2014; Xie et al., 2020). However, another paradoxical immune action was also defined, in which cancer cells induce an immunoediting process causing the immune system to work with the tumor. In this direction, malignant cells change their immune profile to highly express the checkpoint molecules, which protect the normal cells from autoimmunity, leading to tumor immune escape (Facciabene et al., 2012). Discovering this event led up to developing another class of immune-targeting drugs depending on inhibiting the immune checkpoints (Terme et al., 2011; Pardoll, 2012); however, the efficacy of these drugs depends on the degree of infiltrating immune cells, especially T cells (Teng et al., 2015). Even though the immunotherapeutics introduced a clinically applied durable effect in different cancer-bearing patients, a large proportion of patients still had a diminished response (Thorsson et al., 2018). As a result, intensive researches are being applied to understand the tumor immune scene and recognize the predictive markers for the immune therapy-associated response or tolerance (Ganesh et al., 2021; Xing et al., 2021).

The protein kinase C (PKC) family is defined as a group of phospholipid-dependent enzymes that trigger the covalent transfer of phosphate from ATP to serine and threonine domains of proteins. According to their regulation, the PKC family can be categorized into three groups, including nine main isozymes (Finn, 2008) conventional PKCs, PKCα (PRKCA), PKCβ (PRKCB), PKCγ (PRKCG); (Galon et al., 2006) novel PKCs, PKCδ (PRKCD), PKCε (PRKCE), PKCη (PRKCH), PKCθ (PRKCQ); (Zhang et al., 2003) atypical PKCs, PKCι (PRKCI), PKCζ (PRKCZ) (Garg et al., 2014). The PKCs are core elements of various signaling pathways that influence different cellular functions, including proliferation, migration, survival, cell cycle, and polarity (Poole et al., 2004). The PKC isoenzymes were reported with their incorporation in carcinogenesis 3 decades ago, after identifying the PKC as a receptor for the tumor-promoting phorbol esters (Castagna et al., 1982). From that time onwards, many studies have been working to characterize the roles that these molecules play in different cancers (Isakov, 2018). Additionally, several studies discussed the tumor-suppressive role of some PKC isoforms in different cancers (Lu et al., 2009; Dowling et al., 2016). Therefore, in this study, we aimed to explore the differential role of each PKC enzyme in the development of various cancers. Depending on TCGA pan-cancer analysis, we detected the expression profiles of PKC signaling molecules and discussed the effect of methylation and copy number alterations (CNA) on their expression levels. Also, we uncovered the enrollment of PKC family members in regulating the tumor-associated immune cells aggregation with a special reference to the potential value of using the PKC family in predicting the immune landscape and the response to the immunotherapies in different cancers.

## 2 Materials and Methods

### 2.1 Data Sets

All the data used in this study were downloaded from the UCSC Xena database (https://xenabrowser.net/), including transcriptomic profile, methylation levels, GISTIC2_thresholded copy number values, and clinical features of The Cancer Genome Atlas (TCGA) for 33 cancer types. Gene expression levels were presented in log2 (*x* + 1) transformed RSEM normalized count form. The methylation beta values were obtained from the HumanMethylation450 platform. The methylation level of each gene was calculated by the average methylation values of the probes located in the gene promoter region. Since previously proved to be accurate in the TCGA dataset, the overall survival (OS) and progression-free interval (PFI) were used for survival analysis (Araki et al., 2009). GISTIC2 copy number alteration (CNA) scores “−1, 0, +1, +2” represent monoallelic loss, diploid copies, low-level copy gains, and high-level copy amplification, respectively. The homozygous gene deletion scores were excluded from the analysis. To avoid statistical dilution, samples with GISTIC score = “−1” were combined with “0” scored samples, while those with score = “2” were combined with “1” scored samples if the number of any of both was less than 10.

### 2.2 Survival-Related Analyses

Using the R survival package, the association between each PKC gene expression level and survival (OS and PFI) for all cancers was carried out by univariate Cox regression for the high expressed group versus the low one taking the median expression as a cut-off value. The log-rank test was used to compare the difference in survival distribution. Also, the effect of different clinical parameters (age, gender, race) and histological-related features (tumor status, stage, grade) on PKC-linked survival was performed via univariate and multivariate Cox regression analyses; the contentious gene expression values were used for the univariate analysis. PFI analysis for LAML was excluded due to missing data.

### 2.3 Enrichment Analyses

KEGG pathway enrichment analysis was performed using the pathfindR package. While the single-sample gene set enrichment analysis (ssGSEA), which was applied by R package GSVA, was used to investigate the enrichment scores of 28 tumor-infiltrating lymphocytes (TILs) and HLA genes in each cancer type (Charoentong et al., 2017). ESTIMATE algorithm (Yoshihara et al., 2013) was further used to evaluate the immune score referring to the immune cell infiltration level for each separate sample. All immune calculated scores were then correlated with each PKC gene in the TCGA pan-cancers.

### 2.4 Statistical Analyses

All statistical analyses were applied in R programming, version 4.0.3. The Wilcoxon rank-sum test was used to calculate the gene expression and the methylation level differences between cancerous and normal tissues of each cancer type. Also, the effect of each CNA category on the expression level of a particular gene compared to the diploid copies-associated mRNA expression, using the Wilcoxon rank-sum test. The correlation coefficient values were calculated by Spearman’s method. *p*‐values ≤ of 0.05 was considered statistically significant.

## 3 Results

### 3.1 Protein Kinase C Genes Have Heterogeneous Transcriptomic Expression Patterns Between The Cancer Genome Atlas Pan-Cancers

**FIGURE 1:**
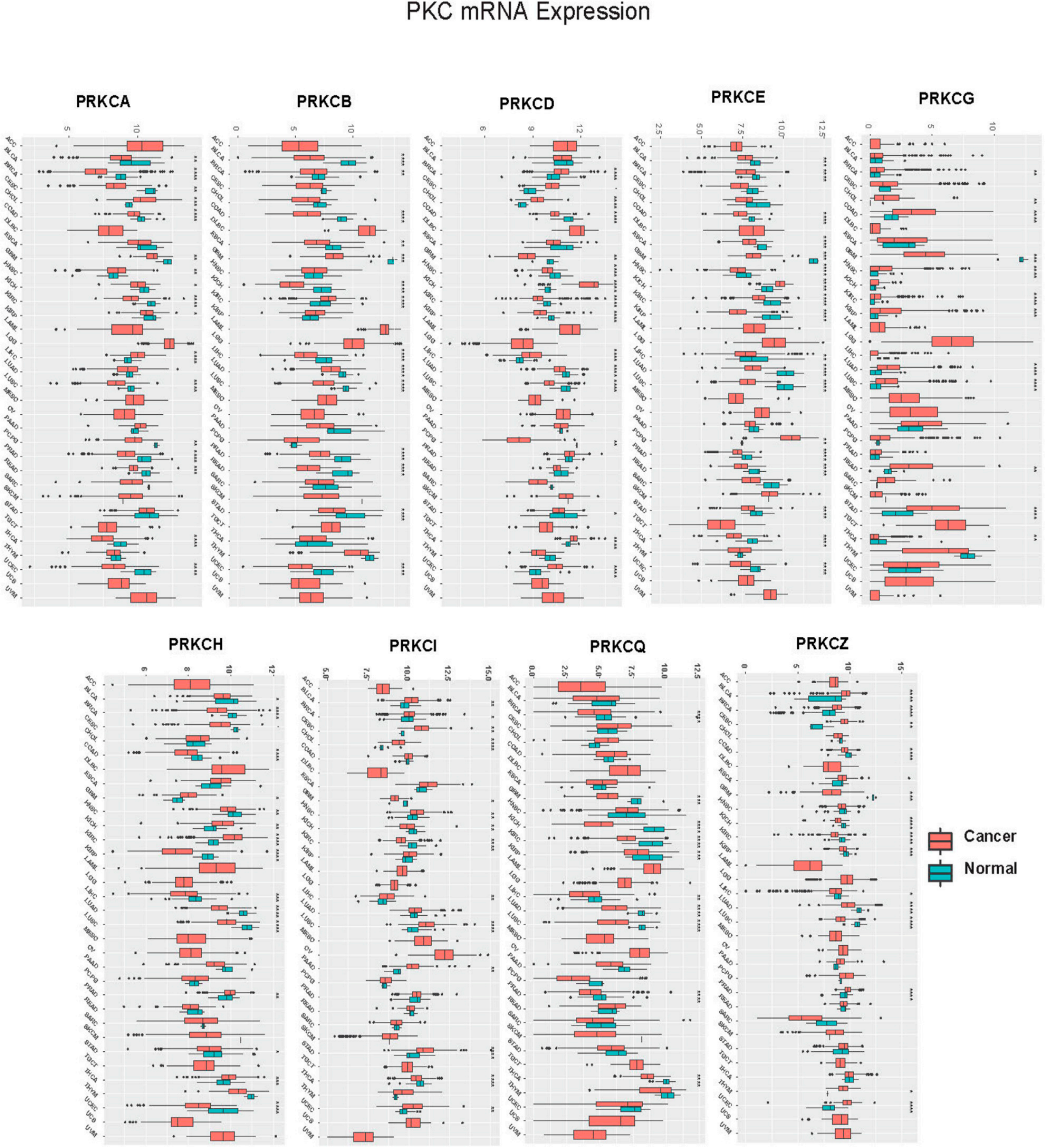
Pan-cancer analysis of PKC mRNA levels between Cancer and Normal in TCGA cancers. *: *p*-value < 0.05; **: *p*-value < 0.01; ***: *p*-value < 0.001.

We performed a pan-cancer analysis to figure out the potential role of PKC family genes in human cancers. Data regarding the TCGA cancer types is presented in Supplementary Table S1. We compared PKC gene expression levels between tumor and adjacent normal tissue in 24 cancer types whose data are available to get a broader view on PKC gene patterns in each cancer. Except for SARC and SKCM, PKC family genes significantly were regulated (either up or down) between cancer and normal tissues with a varying degree (Figure 1; Supplementary Figure S1). Only PRKCQ had a consistent low expression level in cancers compared to normal, including BRCA, GBM, KICH, KIRC, KIRP, LIHC, LUAD, LUSC, PRAD, and THCA, while other genes showed inconsistent expression patterns within different cancer types. PRKCG and PRKCI were both elevated in BRCA, CHOL, HNSC, LUAD, and STAD, in addition to PRKCG higher levels in COAD, KIRC, KIRP, LUSC, and READ, and PRKCI higher levels in BLCA, CESC, LIHC, PAAD, and UCEC. PRKCD was significantly upregulated in BRCA, CESC, CHOL, KICH, LIHC, THCA, and UCEC. In comparison, PRKCZ was raised in 0 BLCA, BRCA, CESC, PRAD, THYM, and UCEC cancerous tissues. In contrast, PRKCH was higher in GBM, KICH, KIRC, PRAD, and THCA tumors. Matched to other genes, PRKCA, PRKCB, and PRKCE were more involved in many normal tissue mRNA profiles than tumor ones. Other significant results were associated with PKC genes’ down-regulation in cancers compared to normal tissues. Collectively, this indicates that PKC family genes may function as oncogenic or oncosuppressor factors depending on the cancer type.

### 3.2 Methylation Level of Protein Kinase C Gene Promoters Correlates Differently With the Expression Level in Pan-Cancers

DNA methylation is an epigenetic process that can significantly modulate gene transcription (Moore et al., 2013). The methylation level of gene promoter was reported to be strongly associated with cancer development (Saghafinia et al., 2018). Promoter hypermethylation frequently induces silencing for oncosuppressor genes, while oncogenes overexpression sometimes occurs under the effect of gene promoter hypomethylation (Ehrlich, 2002; Noguera-Uclés et al., 2020). To understand the mechanism by which each gene of the PKC family is regulated in different cancers, we studied the methylation status of the genes. The data related to CpG islands situated in the promoter regions of PKC family genes are supplied in Supplementary Table S2. We extracted the beta values of CpG sites of PKC genes from the Human Methylation450 platform of TCGA. The methylation level of each gene was calculated by the average methylation of CpG sites in the gene promoter region. We then compared the methylation level of each gene between cancer and adjacent normal tissue in 23 cancer types of TCGA available data.

**FIGURE 2:**
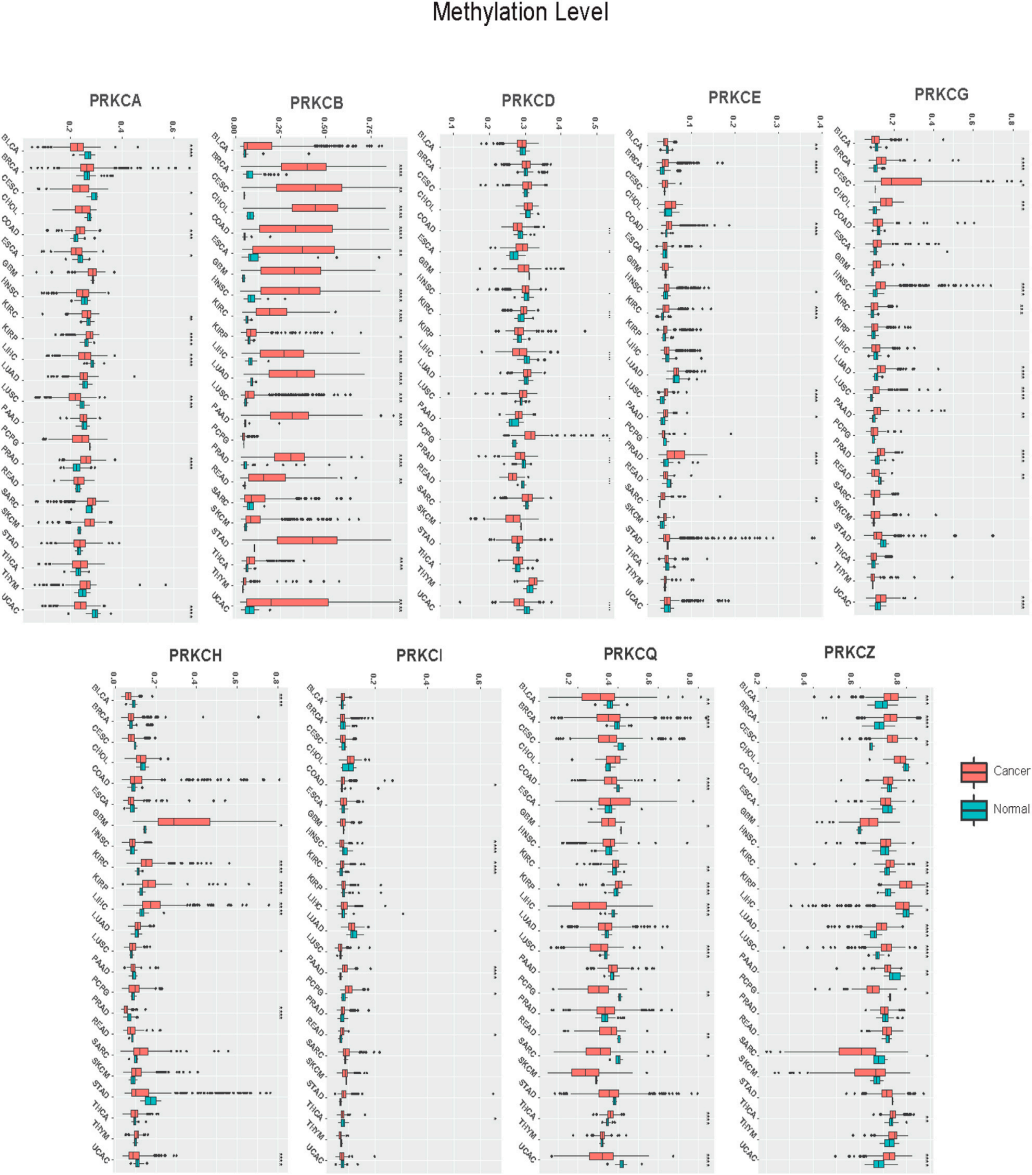
Pan-cancer analysis of PKC genes methylation levels between Cancer and Normal tissues. *: *p*-value < 0.05; **: *p*-value < 0.01; ***: *p*-value < 0.001.

Our results revealed that the methylation pattern of the PKC family genes was variable in different tumors (Figure 2; Supplementary Figure S2). Specifically, PRKCB was consistently hypermethylated in 17 cancer types compared to normal tissue (BRCA, CESC, CHOL, COAD, ESCA, GBM, HNSC, KIRC, KIRP, LIHC, LUAD, LUSC, PAAD, PRAD, READ, THCA, UCEC). Besides, PRKCA was hypomethylated in cancers of BLCA, CESC, CHOL, ESCA, KIRC, LIHC, LUSC, and UCEC and was hypermethylated in COAD, KIRP, and PRAD. Moreover, PRKCD was hypomethylated in COAD, HNSC, LIHC, PRAD, READ, and UCEC, while its methylation level was raised in ESCA, KIRC, LUSC, PAAD, and PCPG tumors. On the other hand, PRKCE methylation was elevated in BRCA, COAD, HNSC, KIRC, LUSC, PAAD, PRAD, and SARC but reduced in BLCA and THCA. PRKCG methylation pattern was mostly elevated in BRCA, CESC, CHOL, HNSC, KIRC, LUAD, LUSC, PAAD, and PRAD, while only lowered in READ. Differently, PRKCH methylation level increased in GBM, KIRC, KIRP, LIHC, and LUSC and decreased in BLCA, PRAD, and UCEC. Further, PRKCI was hypermethylated in COAD, KIRC, PAAD, PCPG, and READ but was hypomethylated in HNSC, LUAD, and THCA. PRKCQ was hypermethylated in BLCA, BRCA, CESC, KIRC, KIRP, LUAD, LUSC, THCA, and UCEC but was hypomethylated in CHOL, LIHC, PAAD, PCPG, and SARC. PRKCZ was mainly hypermethylated in BLCA, BRCA, CESC, KIRC, KIRP, LUAD, LUSC, THCA, and UCEC, while hypomethylated in CHOL, LIHC, PAAD, PCPG, and SARC.

**FIGURE 3:**
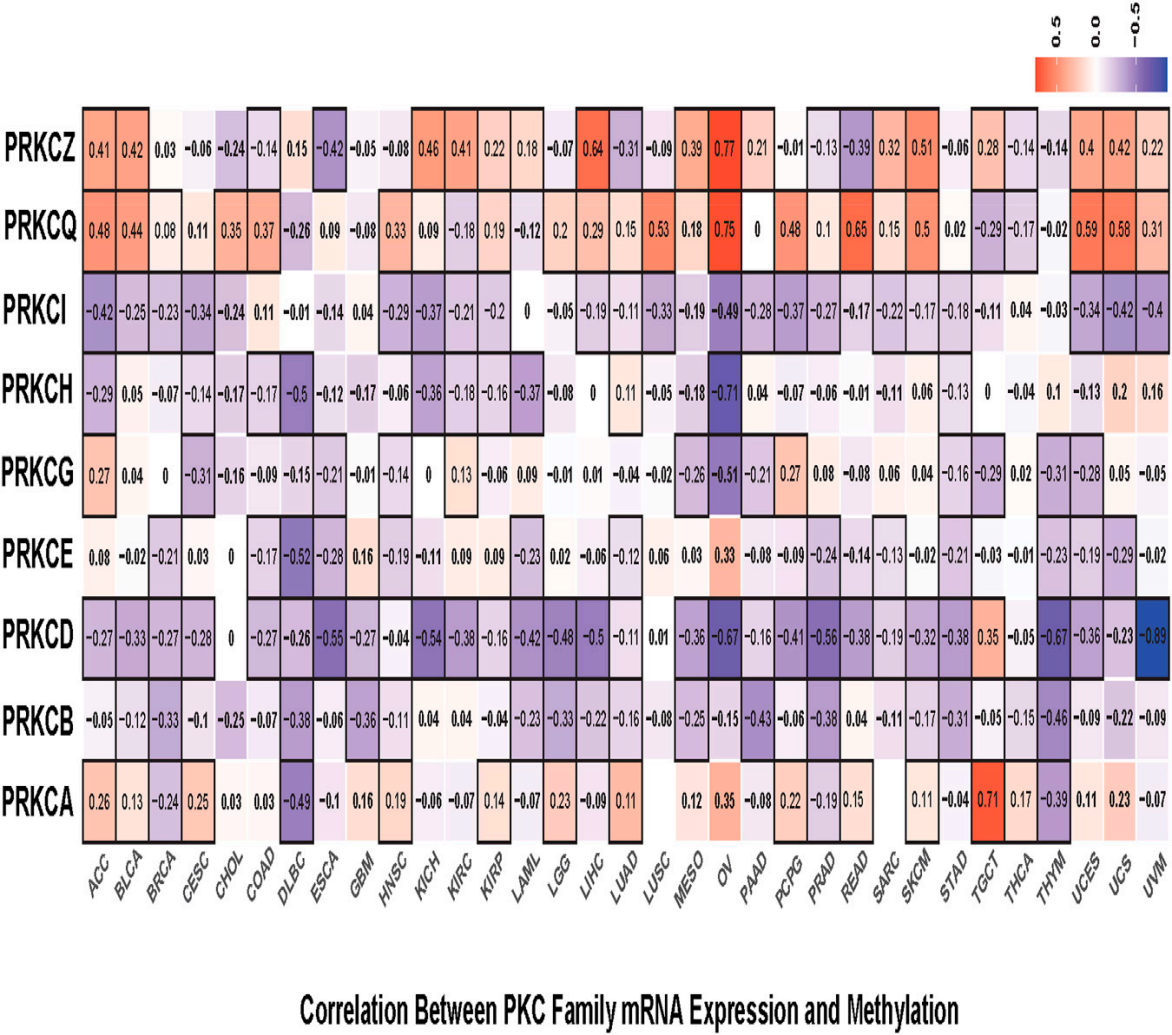
Correlation heatmap between mRNA expression and methylation level of each PKC gene in all TCGA cancers, the color refers to correlation coefficients; the black border refers to the statistically significant correlation (*p*-value < 0.05).

Next, we calculated the correlations between mRNA expression and gene promoter methylation level for each gene in all cancer types. Mostly, PRKCB, PRKCD, PRKCE, PRKCG, PRKCH, and PRKCI showed inverted correlations in many tumors with varying correlation intensity. This suggests that the promoter methylation state of these genes may regulate their expressions among different cancer types. However, PRKCA, PRKCQ, and PRKCZ had positive correlations in many cancer types, with some exceptions shown in Figure 3. Such positive correlations indicate the presence of other regulatory factors that control the expression of these genes.

### 3.3 Protein Kinase C-Associated Copy Number Alterations Contribute to Driving Their Expression in Different Cancer Types

**FIGURE 4:**
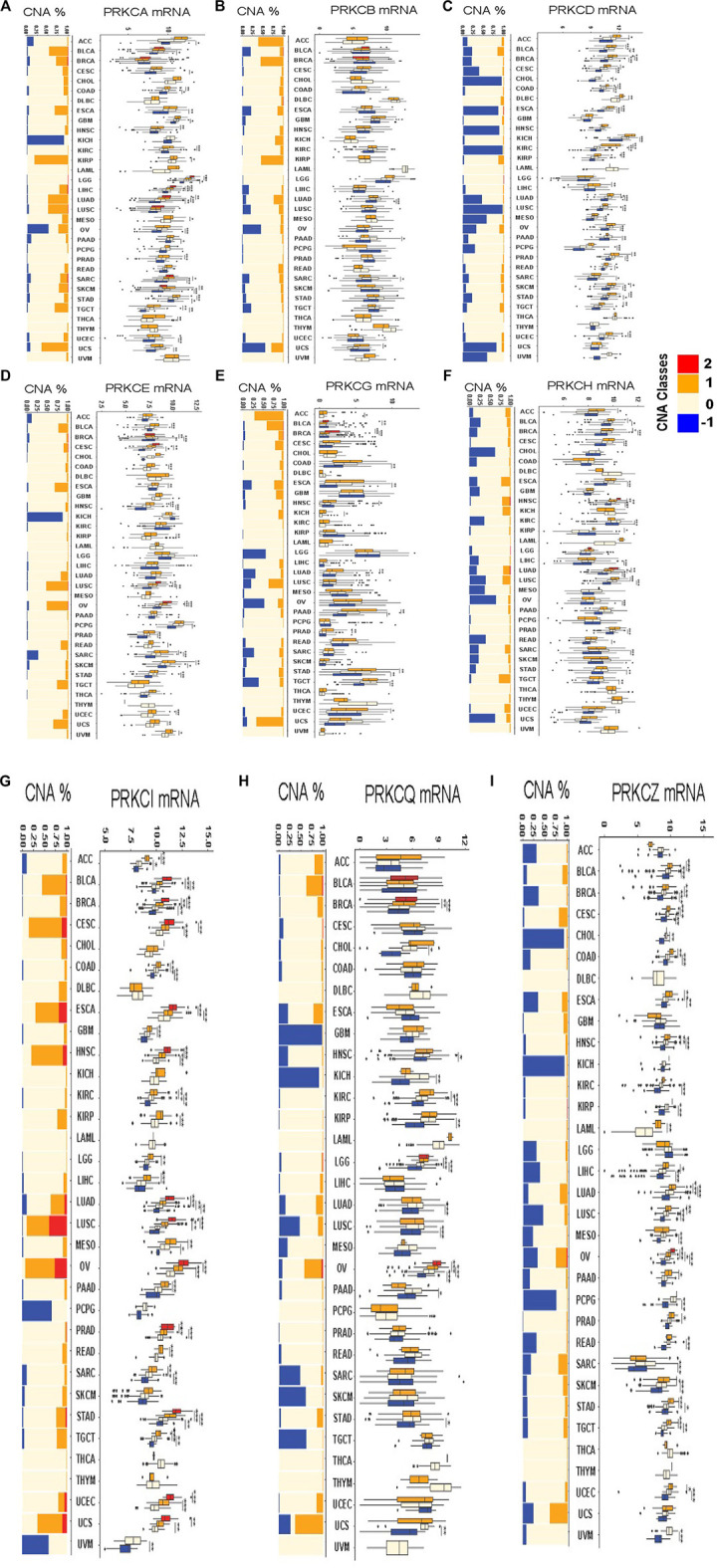
The frequency of copy number alterations and the differences in CNAs-dependent mRNA expressions of each alteration type compared to the diploid copy-associated expression for each PKC gene in all various cancers PRKCA **(A)**, PRKCB **(B)**, PRKCD **(C)**, PRKCE **(D)**, PRKCG **(E)**, PRKCH **(F)**, PRKCI **(G)**, PRKCQ **(H)**, PRKCZ **(I)**. The red color = high-level copy amplification, the orange color = low-level copy gains, the light color = diploid copies, the blue color = monoallelic loss. *: *p*-value < 0.05; **: *p*-value < 0.01; ***: *p*-value < 0.001.

To understand the underlying mechanisms that regulate PKC gene expressions at the genetic level, we explored the effect of CNAs on the expression levels of the genes in different cancer types. First, we evaluated the frequency of copy number gains (CNGs = high-level copy amplification and/or low-level copy gains) and loss (monoallelic loss) in all tumor types in the TCGA database using the GISTIC scores (+2, +1, 0, −1). GISTIC score “+2” was rarely observed in PKC family genes, except for PRKCI, where it was associated with nearly 10–30% high-level CNG in CESC, ESCA, HNSC, LUAD, LUSC, UCEC, and UCS (Figures 4A–I). The monoallelic loss was most prevalent in PRKCD, PRKCH, PRKCZ, and PRKCQ across different cancer types. While low-level CNGs were frequently observed in PRKCA, PRKCB, PRKCE, PRKCG, and PRKCI. Besides, PRKCD, PRKCH, PRKCZ, and PRKCQ were associated with varying degrees of low-level CNGs along with different cancers.

Next, we evaluated the effect of PKC-associated CNAs on driving the gene expression in various cancer types by comparing the mRNA level of each alteration type with the mRNA level of diploid copies of the same gene. For PRKCI, except in DLBC, KICH, LAML, THCA, and THYM, all tumors with CNGs acquired elevated mRNA gene expression, and most copy number losses were linked to a reduction in mRNA level. Also, in PRKCZ, all monoallelic losses caused reduced mRNA expression, and most CNGs elevated the gene expression, except in ACC, GBM, KICH, LGG, THCA, THYM, and UCS. Many CNAs in PRKCD regulated their mRNA expression, except in GBM, HNSC, LAML, LGG, THYM, and UCS. The combined CNAs classes in PRKCA regulated the expression in only COAD, KIRP, LGG, LIHC, LUAD, MESO, STAD, and TGCT. While PRKCE CNAs affected the gene level in CESC, COAD, HNSC, KIRP, LUSC, OV, SARC, SKCM, UCEC, UCS, and UVM. Besides, PRKCH expression was regulated by CNAs in BLCA, BRCA, HNSC, LUAD, and LUSC; and CNAs controlled PRKCG expression in ACC, PAAD, and STAD; CNAs also guided PRKCQ expression in KIRC, LGG, and OV.

### 3.4 Protein Kinase C Genes Have a Prognostic Value in The Cancer Genome Atlas Pan-Cancers

**FIGURE 5:**
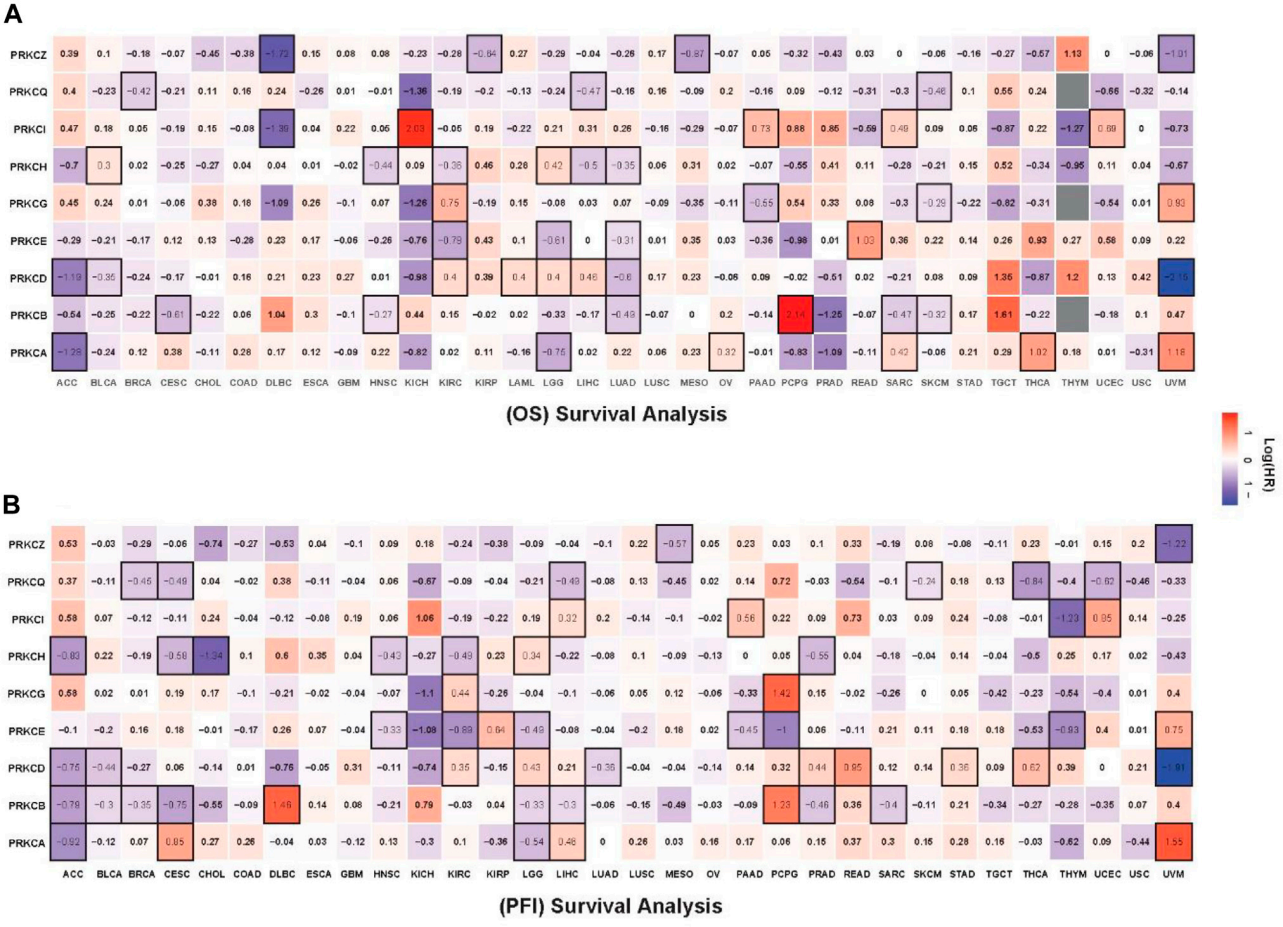
Survival heatmap for OS **(A)**, PFI **(B)** analysis comparing the HR of high expressing group versus low expressing group for all PKC genes in the TCGA tumors. The red color indicates HR for the high-expressing group; the blue color indicates the HR for the low-expressing group; the black border refers to the statistically log-rank test (*p*-value < 0.05).

We studied the effect of all PKC family gene expressions on the survival in TCGA cancers by dividing the patients in each tumor, according to the tested gene expression, into high and low groups, using the median gene expression value as a cut-off. We then calculated the hazard ratio (HR) of OS and PFI for the high group versus the low one, and the log-rank test was used to detect the difference in the survival time. Combining the significant results of OS and PFI analyses, we found that the high-PRKCA expressing group had a better survival rate in ACC and LGG, while the low-PRKCA expressing group had better survival in CESC, LIHC, OV, SARC, THCA, and UVM (Figures 5A,B). We also observed that high PRKCB prolonged the survival time in ACC, BLCA, BRCA, CESC, HNSC, LGG, LIHC, LUAD, PRAD, SARC, and SKCM patients, but shortened the survival time in DLBC and PCPG. Additionally, high PRKCD enhanced patients’ survival rate with ACC, BLCA, LUAD, and UVM but worsened patients’ survival with KIRC, LAML, LGG, LIHC, PRAD READ, STAD, and THCA. On the other hand, high PRKCE elevated the survival rate in HNSC, KIRC, LGG, LUAD, PAAD, PCPG, and THYM but lowered the survival rate in KIRP, READ, and UVM. High PRKCG improved PAAD and SKCM patients’ survival and reduced KIRC, PCPG, and UVM patients’ survival. Furthermore, elevated PRKCH was associated with better survival in ACC, CESC, CHOL, HNSC, KIRC, LIHC, LUAD, and PRAD, but worsened BLCA and LGG patients’ prognosis. Moreover, PRKCI expression was directly related to the survival rate in DLBC and THYM and inversely related to the survival rate in KICH, LIHC, PAAD, SARC, and UCEC. In contrast, patients with low PRKCQ expression had lower survivals in BRCA, CESC, LIHC, SKCM, THCA, and UCEC. While, the high expression level of PRKCZ seemed to improve the survival rate in patients with DLBC, KIRP, MESO, and UVM.

### 3.5 Protein Kinase C-Linked Survival Associations are Mostly Independent of Multivariate Clinical Characteristics

*Via* univariate and multivariate Cox regression analysis of OS and PFI, we estimated the association of PKC family genes expression, as contentious values, with different clinical (age, gender, race) and histological-related features (tumor status, stage, and grade) in determining patients’ prognosis in each tumor type. Supplementary Table S3 presents only the data of significant PKC-linked survival as univariate compared to that after being combined with other variants. We found that most of the significant PKC-linked survival associations were independent of the tested clinical characteristics. After using both the clinical and histological-related features, PKC genes were independently affecting patients’ prognosis in many cancers as the following:

PRKCA was independently affecting survival in patients with HNSC, LGG, MESO, and SKCM. In comparison, PRKCB was independently controlling the prognosis of patients with CESC, HNSC, LGG, LIHC, LUAD, PCPG, and SARC. Conversely, ACC, BLCA, LGG, LIHC, LUAD, READ, and UVM patients’ survival pertained to the PRKCD level. On the other hand, KIRC, LGG, LUAD, and UVM fate significantly particularized by PRKCE. But, PRKCG level only selectively influenced the prognosis in patients with ACC, KIRC, and PCPG. Furthermore, PRKCH high level independently prolonged the survival time in patients with BRCA, CESC, HNSC, KIRC, LIHC, and LUAD. In contrast, PRKCI high expression strictly shortened the survival time in patients with KICH and PAAD without external effect from the multivariate tested features. Also, KICH and LIHC patients’ survival was solely associated with PRKCQ level, and PRKCZ level significantly affected BRCA, LGG, LUAD, and MESO patients’ prognosis.

### 3.6 Protein Kinase Cs-Correlated Genes are Highly Enriched in Immune and Tumor‐Related Cellular Signaling Pathways in Most Tumor Types

**FIGURE 6:**
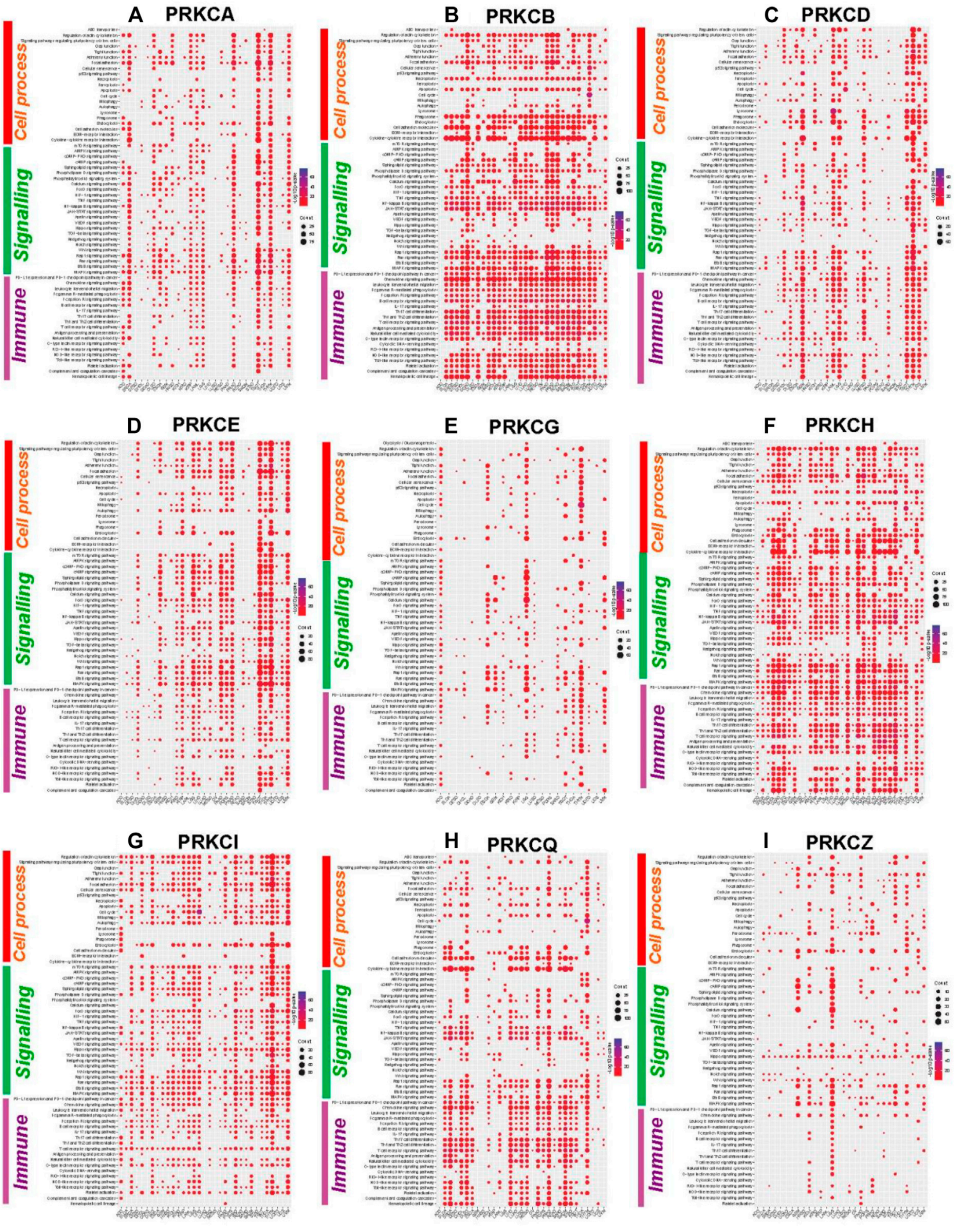
KEGG pathways enrichment analysis of PKC significantly correlated genes in different cancers: PRKCA **(A)**, PRKCB **(B)**, PRKCD **(C)**, PRKCE **(D)**, PRKCG **(E)**, PRKCH **(F)**, PRKCI **(G)**, PRKCQ **(H)**, PRKCZ **(I)**. The dot size refers to the number of enriched genes in the pathway, while the color refers to the adjusted *p*‐value.

To explore the role of the PKC family in different cancers, we first detected the genes correlating with each PKC isoform in different cancers using Spearman’s method. Then, we selected the genes with a correlation coefficient equal to or more than 0.4 and with an adjusted *p*-value < 0.05 to be used as significantly correlated genes in KEGG pathway enrichment analysis. Our results showed that PKCs significantly correlated genes were principally enriched in immune and tumor‐related cellular signaling pathways in most tumor types (Figure 6). Of the immune-related pathways, PD-L1 expression and PD-1 checkpoint pathway were highly correlated with all PKC genes among most cancers except PRKCZ. Other enriched pathways were also linked to PRKCB, PRKCH, and PRKCQ in most cancers and to PRKCA, PRKCD, and PRKCE in some cancers including, Toll-like receptor signaling pathway, NOD-like receptor signaling pathway, Natural killer cell-mediated cytotoxicity, Th1 and Th2 cell differentiation, Th17 cell differentiation, B cell receptor signaling pathway, and Leukocyte transendothelial migration.

On the other hand, many tumor‐related cellular signaling pathways were linked to PKC genes in most tumor types. These pathways involved MAPK signaling pathway, Ras signaling pathway, Wnt signaling pathway, Notch signaling pathway, Hedgehog signaling pathway, TGF-beta signaling pathway, Hippo signaling pathway, cAMP signaling pathway, and mTOR signaling pathway.

### 3.7 Protein Kinase Cs are Biomarkers for Tumor Immune Landscape and the Response to Immunotherapies

**FIGURE 7:**
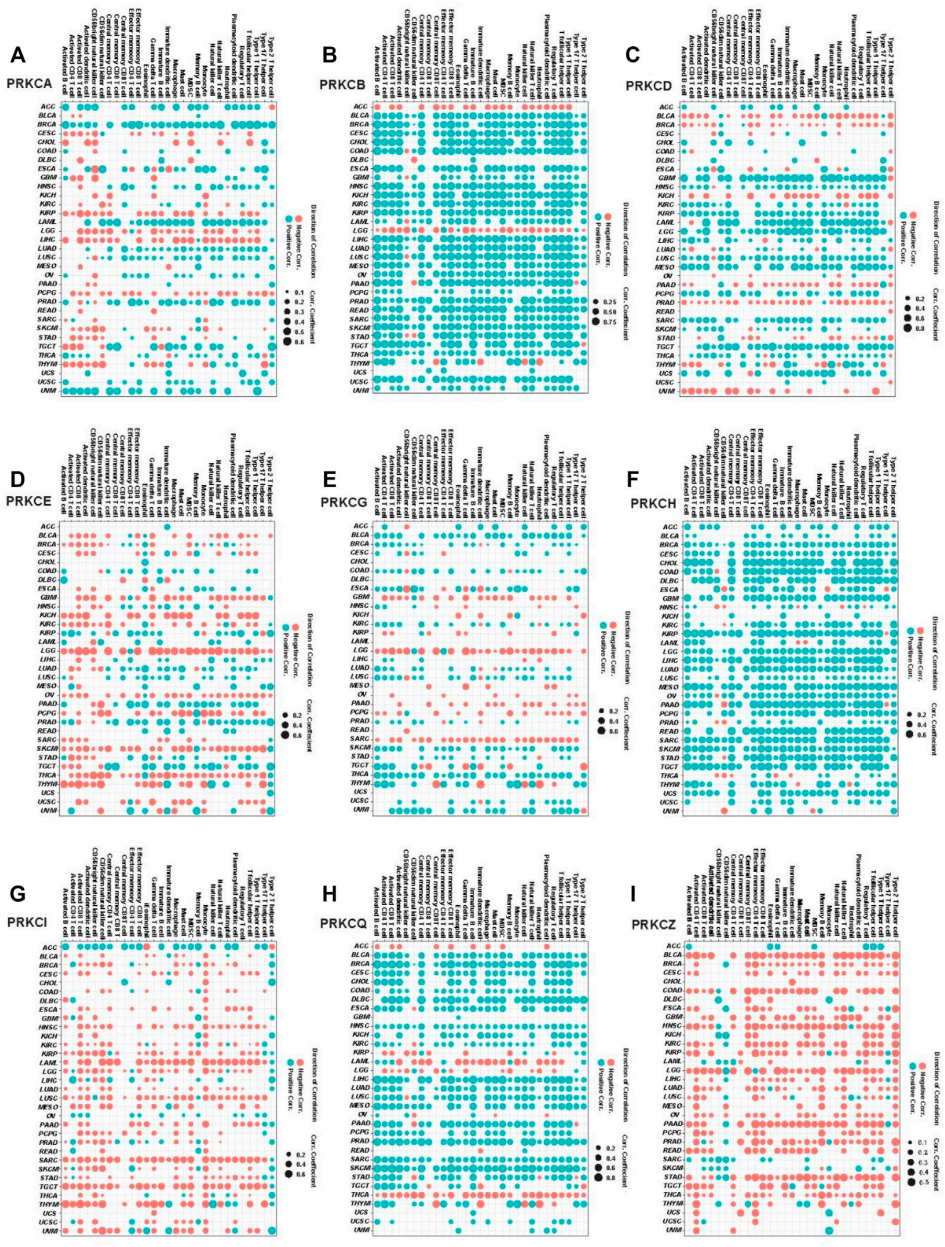
Pan-cancer analysis for the significant correlation between PKC genes expressions and ssGSEA scores of 28 immune cell subpopulations; PRKCA **(A)**, PRKCB **(B)**, PRKCD **(C)**, PRKCE **(D)**, PRKCG **(E)**, PRKCH **(F)**, PRKCI **(G)**, PRKCQ **(H)**, PRKCZ **(I)**. The dot size states the correlation coefficients, while the color refers to the direction of the correlation, blue = positive correlation, and red = negative correlation (*p*-value < 0.05).

The later pathways enrichment results urged us to deeply mine the role of the PKC family in tumor immunity. We used ssGSEA to calculate the composite expression of signatures related to 28 TILs for all cancer types (Charoentong et al., 2017). Then, we calculated the correlation coefficients between the immune scores and PKC gene expressions in each cancer. As shown in Figure 7, PRKCI and PRKCZ expressions were negatively correlated with most TILs in most cancer types (except PRKCI in ACC and OV; PRKCZ in ACC, SARC SKCM, had a positive correlation). In contrast, PRKCB, PRKCH, and PRKCQ expressions were positively correlated with TLLs among most cancer types. Otherwise, the correlations between PRKCA, PRKCD, PRKCE, and PRKCG expressions and the infiltration of the immune cells varied between cancer types. For example, PRKCA in BRCA, LAML, and UVM; PRKCD in GBM, LAML, LGG, and MESO; PRKCE in PRAD; PRKCG in BLCA and THCA all were positively associated with TILs. On the other hand, PRKCA in KIRP, LGG, and LIHC; PRKCD in BLCA, KICH, and PRAD; PRKCE in LGG, PCPG, SKCM, and THCA; PRKCG in LGG and SARC were negatively correlated with TILs.

Besides, we applied the ESTIMATE method to calculate the immune score for each sample in all cancer types, followed by measuring the correlation coefficient between the estimated scores and PKC family expressions. We found that nearly most of the results were similar to that of ssGSEA (Supplementary Figure S3).

**FIGURE 8:**
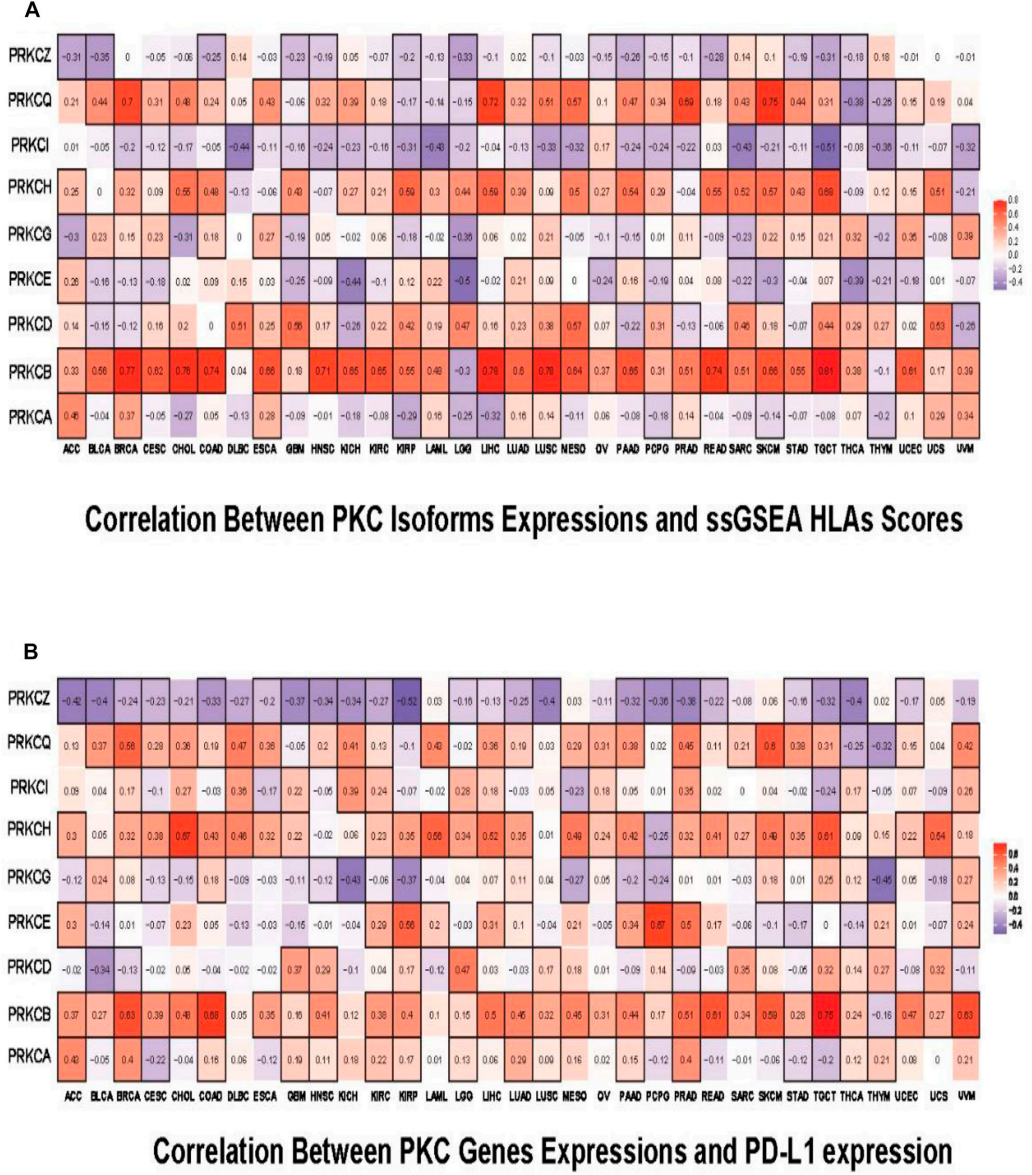
Pan-cancer predictive analysis for the effect of PKC family on patient’s response to immunotherapies. **(A)** Correlation of PKC isoforms expressions and ssGSEA HLAs scores. **(B)** Correlation of PKC genes expressions and PD-L1 expression. The color refers to correlation coefficients, and the black border refers to the statistically significant correlation (*p*-value < 0.05).

We then tested the probability that PKC gene levels can affect cancer patient’s response to the immunotherapies. By using ssGSEA, we inspected the correlation between the expression levels of PKC genes and the HLA genes enrichment scores. HLAs are cell-surface proteins encoded by a group of MHC genes and functions to present the cancer cells to immune cells. Many immunotherapies are HLA dependent such as TILs therapy and TCR-engineered T cells (TCR-Ts) therapy (Wang et al.). We found that PRKCB, PRKCH, and PRKCQ levels were directly associated with HLAs scores in almost most cancers, especially for PRKCB, whose elevated expression moderately to strongly controlled the HLA level in 28 cancer types (*R* ˃ 0.3) (Figure 8A). For PRKCA, PRKCD, PRKCE, and PRKCG, the correlation between their expressions and HLAs enrichment scores were variable among different cancers. However, the direction of this correlation followed the same pattern of gene expression-linked TILs in each cancer. In contrast, PRKCI and PRKCQ were generally inversely related to HLAs presence. Besides, PD-L1 expression is a predictive biomarker for the response to PD-1/PD-L1-directed immunotherapy (Aguiar et al., 2017), a recently designed immunotherapy that has been applied in various tumors and exhibited some promising effects (Iwai et al., 2002; Ott et al., 2013). Using correlation analysis between PD-L1 and PKC family expressions, we found that mostly PKC isoforms were directly correlated with PD-L1 level, except for PRKCQ, whose expression consistently had an inverted correlation with PD-L1 level in 23 tumors (Figure 8B).

## 4 Discussion

Immunotherapy is a successful cancer treatment; scientists scored it number one of the ten most significant scientific breakthroughs in 2013. However, understanding the tumor immune landscape is a key effector on the patients’ response to immunotherapeutic (Thorsson et al., 2018; Torphy et al., 2020). Hence, recognizing tumor immune-regulatory factors and explaining the underlying molecular process is essential to improve cancer patients’ prognosis and direct the production of successful combination therapies (Feng et al., 2021; Yang et al., 2021). In this study, we focused on the PKC family members, a group of cell signaling regulatory proteins, to figure out their enrollment in the tumorigenesis, with a special reference to their association to the tumor immune response. Due to their function as kinases and their characterization as the direct targets of phorbol esters, important tumor-promoting molecules, PKC isoforms have long been considered oncoproteins (Castagna et al., 1982). Nevertheless, recent researches have debated that the activation of some PKC isoforms antagonizes the carcinogenic process with many queries about the precise mechanism (Antal et al., 2015; Newton, 2018; Parker et al., 2020).

Using TCGA data pan-cancer analysis, we investigated the fundamental PKC-related changes in the organization of different cancers. We noticed significant variations in the PKC gene expression levels between cancer and normal tissues, and these variations were associated with patients’ prognosis in many tumors. Collectively, the expression levels of PRKCI and PRKCG were elevated in most of the significantly compared cancers, while PRKCA, PRKCB, PRKCE, and PRKCQ tended to be downregulated among most cancer types. However, some cancers showed a lowered level of PRKCI (GBM, KICH, HIRC, THCA) and PRKCG (GBM, THCA). Other gene patterns were wildly inconsistent with changing the tissue type. The heterogeneous patterns of PKC gene expressions between different types of cancer suggest that PKC regulation might be tissue-specific. Similar divergencies in the PKC profile in different cancers were previously reported (Garg et al., 2014). However, there are some researches that described contrary differential PKC expressions between cancer and normal tissues. For instance, PRKCE was previously described to be abnormally overexpressed in non-small cell lung cancer (NSCLC) (Bae et al., 2007). Also, in colon cancer, PRKCB level was reported to be either elevated, lowered or not changed (Isakov, 2018). Furthermore, PRKCI predominantly behaved as an oncogene whose elevation was associated with the induction of different tumors (Fields and Regala, 2007). Collectively, all findings ensured the involvement of PKCs in one or more ways in different cancers.

Besides, univariate and multivariate COX analyses showed that the significant influence of the expression of PKC genes on overall or progression-free survival in most cancers was independent of the tested features (age, gender, race). Where, even after ignoring the impact of these variables, the relation was still significant. On the other hand, extra adding of the histological-related features to the multivariate analysis, reliant on gene expression, proved that patients’ survival of HNSC, LGG, MESO, and SKCM depended on PRKCA expression, while that of CESC, HNSC, LGG, LIHC, LUAD, PCPG, and SARC was dependent on PRKCB. Additionally, patients’ prognosis in ACC, BLCA, LGG, LIHC, LUAD, READ, and UVM attributed to PRKCD; and that of KIRC, LGG, LUAD, and UVM referred to PRKCG; but PRKCG level only selectively influenced the prognosis in patients with ACC, KIRC, and PCPG. Furthermore, PRKCH high level dependently prolonged the survival time in patients with BRCA, CESC, HNSC, KIRC, LIHC, and LUAD. In contrast, PRKCI high expression singly shortened the survival time in patients with KICH and PAAD without external effect from the multivariate tested features. Also, KICH and LIHC patients’ survival was associated with PRKCQ level, and PRKCZ level significantly affected BRCA, LGG, LUAD, and MESO patients’ prognosis severally. Such results potentiate the theory of using PKC as prognostic biomarkers in many cancers of different origins.

Furthermore, we observed a significant variation in the methylation level of the promoters of PKC members between cancer and normal tissue in many cancers. This variation was mostly negatively correlated with the gene expression level, especially of PRKCB, PRKCD, PRKCE, PRKCG, PRKCH, and PRKCI. Such observation was consistent with the fact that gene promoter methylation inversely correlates with its expression (Bird, 2002; Jin and Robertson, 2013). However, many strong positive correlations were also observed, principally associating to PRKCQ and PRKCZ. This indicates the presence of other regulatory factors involved during PKC family expression. Moreover, like mRNA expression, some inconsistent figures of the methylation pattern of PKC genes between different tumors support the observation that PKC family organization depends on the cell type context.

CNAs are a robust gene expression regulatory factor in cancer (Stranger et al., 2007; Henrichsen et al., 2009; Tang and Amon, 2013). Firstly, we found that all the alteration types existed in PKC isoforms within most cancers after excluding the homozygous gene deletion from the analysis. CNGs proportions were higher in PRKCI and frequently observed in PRKCA, PRKCB, PRKCE, and PRKCG. The monoallelic loss was most prevalent in PRKCD, then PRKCH, PRKCZ, and PRKCQ across many cancer types compared to other genes. Besides, PRKCD, PRKCH, PRKCZ, and PRKCQ were associated with varying degrees of low-level CNGs along with different cancers. Some previous studies reported similar alterations affecting some PKC genes in different cancers (Zhang et al., 2006; Liu et al., 2020).

Depending on our results, PRKCI, PRKCD, and PRKCZ associated-CNAs were driving their expressions in most cancers (except for PRKCI in DLBC, KICH, LAML, THCA, and THYM; PRKCD in GBM, HNSC, LAML, LGG, THYM, and UCS; and PRKCZ in ACC, GBM, KICH, LGG, THCA, THYM, and UCS). Other genes showed a varying degree of CNAs-induced transcription regulation. Besides, this association was significant even with a low alteration proportion in many cancers.

Since PRKCI was overexpressed in many cancer types, it had a high proportion of CNGs, and these gains were strongly associated with PRKCI expression so that CNGs might be the primary up-regulatory mechanism of PRKCI in diverse cancer types. These results match with the fact that PRKCI is located in the 3q26 chromosomal region, one of the most commonly gained genomic sites in cancer. In this portion, CNG occurs in more than 20% of all human cancers and is usually associated with poor patient prognosis, indicating a direct role for the amplified genes within this region in promoting tumorigenesis (Fields et al., 2016).

In addition to cell signaling and cellular processes-related pathways that were previously expected, the pathway enrichment analysis results exhibited that the PKC correlated genes were enriched in many immune-related pathways. For example, PD-L1 expression and PD-1 checkpoint pathway, Toll-like receptor signaling pathway, NOD-like receptor signaling pathway, Natural killer cell mediated cytotoxicity, Th1 and Th2 cell differentiation, Th17 cell differentiation, B cell receptor signaling pathway, and Leukocyte transendothelial migration, all were enriched with increased PKCs expression depending on the cancer type.

Several studies have previously described the integration of PKC members in regulating immune cells. PRKCB, as an example, induced NF-kappa B-mediated B cell receptor (BCR)-activation (Su et al., 2002). Also, PRKCB was identified as one of the main immune-linked indexes which stimulate TILs in NSCLC (Pang et al., 2020). PRKCI, in contrast, increased myeloid-derived suppressor cells and decreased cytotoxic T-cell infiltration in ovarian cancer, leading to an immune-suppressive TME (Sarkar et al., 2017). In addition, PRKCA is crucial for IFNγ production by T cell and for IgG2a/b class switching of B cell (Pfeifhofer et al., 2006). Furthermore, PRKCQ stimulates tumor immune recognition by inducing T cell receptor (TCR) pathways and inhibiting Treg cells (Kwon et al., 2012; He et al., 2014). In this study, an important finding was that all PKC kinases correlated with the tumor immune cells infiltration in most cancers. By studying the relationship between the activity of PKC genes and the signatures of various immune cell subpopulations, we reported that in most cancer types, the levels of PRKCB, PRKCH, and PRKCQ were positively associated with the infiltration of most immune cells. In comparison, PRKCI and PRKCZ expression levels were negatively associated with the recruitment of multiple immune cells. Whereas PRKCA, PRKCD, or PRKCG expression levels were negatively associated with the infiltration of immune cells in some cancers and positively correlated with immune infiltration in other cancers. All these results ensure the importance of all PKC members as tumor immune biomarkers and to understand the immune landscape of different cancers.

The high levels of HLA genes and PD-L1 levels were frequently used as predictive biomarkers for good patient’s responses to several immune therapies (Aguiar et al., 2017; Hurkmans et al., 2020; Anderson et al., 2021). Correlating PD-L1 level and the collective HLA genes signature to PKC family expressions revealed that elevated PRKCB, PRKCH, or PRKCQ might improve the patients’ response to HLA-dependent or PD-L1 blocking-dependent immunotherapies. On the other hand, PRKCI elevated level was generally predicted to enhance the response to PD-L1 blocking-dependent immunotherapies in many cancers (except for ESCA, OV, and TGCT, in which PRKCI level was inversely correlated with PD-L1). At the same time, a high level of PRKCI was predictive of inadequate response to HLA-dependent immunotherapies in most cancers. Furthermore, elevated PRKCZ levels generally might lower the response to immunotherapies in almost all cancers due to the inverted correlation with TILs, HLA genes, and PD-L1 expression. Also, we predicted that PRKCA, PRKCD, and PRKCE might be involved in improving the response of many cancers to PD-L1 blocking-dependent immunotherapies, but their effect on HLA-dependent therapies may run in line with their effect on the TILs aggregation. For example, high PRKCD in GBM, LGG, and TGCT was linked to elevated immune cells infiltration and HLA genes enrichment level, but elevated PRKCE in LGG and KICH was associated with inhibition of TIL and low HLA scores.

It should be noted that this study has some limitations. We depended on correlation analysis between the TILs signatures and the PKC expressions to predict the role of PKCs in the recruitment of immune cells in the tumor microenvironment (TME). However, TME is heterogeneous and composed of different types of cells, including cancer cells, vascular endothelial cells, cancer-associated fibroblast (CAF), and TILs. To differentiate the specific contribution of PKC from each cell type of TME, further experimental validations with immunohistochemistry or single-cell RNA sequencing techniques are required.

In conclusion, the combined features of PKC members not only can give a prognostic picture of cancer fate but also a strong understanding of the tumor immune microenvironment pattern and can be used to predict the response to many immune therapeutics. Since all PKC members are involved in tumor immune regulation, further studies are required to uncover the elaborated mechanisms and to check if there is a co-operative PKC inter-family interaction toward the tumor immune modulation.

## Data Availability

The original contributions presented in the study are included in the article/Supplementary Material, further inquiries can be directed to the corresponding authors.
