# Supplementary material for: Pan-Cancer Study on Protein Kinase C Family as a Potential Biomarker for the Tumors Immune Landscape and the Response to Immunotherapy
Source: Front Cell Dev Biol. 2022 Jan 31;9:798319. doi: 10.3389/fcell.2021.798319 (PMC8841516; doi:10.3389/fcell.2021.798319)

**Pan-cancer study on protein kinase C family as a potential biomarker for the tumors immune landscape and the response to immunotherapy**

**Supplementary tables:**

**Table S1 Cancer names and sample numbers of TCGA dataset.**

| **Cancer Name** | **Cancer Type** | **mRNA (N-T)** | **Methylation (N-T)** | **CAN**  **(T)** |
| --- | --- | --- | --- | --- |
| **Adrenocortical carcinoma** | ACC | 0-79 | 0-80 | 90 |
| **Bladder urothelial carcinoma** | BLCA | 19-408 | 21-413 | 408 |
| **Breast invasive carcinoma** | BRCA | 114-1104 | 98-790 | 1080 |
| **Cervical and endocervical cancers** | CESC | 3-305 | 3-309 | 295 |
| **Cholangiocarcinoma** | CHOL | 9-36 | 9-36 | 36 |
| **Colon adenocarcinoma** | COAD | 41-288 | 3-299 | 451 |
| **Lymphoid Neoplasm Diffuse Large B-cell Lymphoma** | DLBC | 0-48 | 0-48 | 48 |
| **Esophageal carcinoma** | ESCA | 11-185 | 16-186 | 184 |
| **Glioblastoma multiforme** | GBM | 5-167 | 2-153 | 577 |
| **Head and Neck squamous cell carcinoma** | HNSC | 44-522 | 50-530 | 522 |
| **Kidney Chromophobe** | KICH | 25-66 | 0-66 | 66 |
| **Kidney renal clear cell carcinoma** | KIRC | 72-534 | 160-320 | 528 |
| **Kidney renal papillary cell carcinoma** | KIRP | 32-291 | 45-276 | 288 |
| **Acute Myeloid Leukemia** | LAML | 0-173 | 0-194 | 191 |
| **Brain Lower Grade Glioma** | LGG | 0-530 | 0-530 | 513 |
| **Liver hepatocellular carcinoma** | LIHC | 50-373 | 50-379 | 370 |
| **Lung adenocarcinoma** | LUAD | 59-517 | 32-460 | 516 |
| **Lung squamous cell carcinoma** | LUSC | 51-502 | 43-372 | 501 |
| **Mesothelioma** | MESO | 0-87 | 0-87 | 87 |
| **Ovarian serous cystadenocarcinoma** | OV | 0-308 | 0-10 | 579 |
| **Pancreatic adenocarcinoma** | PAAD | 4-179 | 10-185 | 184 |
| **Pheochromocytoma and Paraganglioma** | PCPG | 3-184 | 3-184 | 162 |
| **Prostate adenocarcinoma** | PRAD | 52-498 | 50-499 | 492 |
| **Rectum adenocarcinoma** | READ | 10-105 | 7-99 | 165 |
| **Sarcoma** | SARC | 2-263 | 4-265 | 257 |
| **Skin Cutaneous Melanoma** | SKCM | 1-473 | 2-474 | 367 |
| **Stomach adenocarcinoma** | STAD | 35-415 | 2-393 | 441 |
| **Testicular Germ Cell Tumors** | TGCT | 0-156 | 0-156 | 150 |
| **Thyroid carcinoma** | THCA | 59-513 | 56-515 | 500 |
| **Thymoma** | THYM | 2-120 | 2-124 | 123 |
| **Uterine Corpus Endometrial Carcinoma** | UCEC | 24-201 | 46-432 | 539 |
| **Uterine Carcinosarcoma** | UCS | 0-57 | 0-57 | 56 |
| **Uveal Melanoma** | UVM | 0-80 | 0-80 | 80 |

**Table S2 CpG Propes of PKC Promoters.**

| **CpG Sites of PKC Promoters** | | | | | | | | | |
| --- | --- | --- | --- | --- | --- | --- | --- | --- | --- |
| CpG site name | PKC genes | CpG site name | PKC genes | CpG site name | PKC genes | CpG site name | PKC genes | CpG site name | PKC genes |
| cg00050312 | PRKCA | cg23460210 | PRKCG | cg01164686 | PRKCZ | cg11345323 | PRKCZ | cg20250605 | PRKCZ |
| cg03055499 |  | cg25103043 |  | cg01970427 |  | cg11781622 |  | cg20975074 |  |
| cg03371918 |  | cg26626089 |  | cg02138098 |  | cg11796233 |  | cg21137823 |  |
| cg04533189 |  | cg27552287 |  | cg02293396 |  | cg11835265 |  | cg22174681 |  |
| cg09645572 |  | cg00012992 | PRKCH | cg02300356 |  | cg12085265 |  | cg22332339 |  |
| cg17921248 |  | cg00244040 |  | cg02361803 |  | cg12639453 |  | cg22342249 |  |
| cg21350778 |  | cg02121330 |  | cg02482150 |  | cg12711627 |  | cg22366001 |  |
| cg00735962 | PRKCB | cg02282237 |  | cg02587648 |  | cg12713909 |  | cg22626579 |  |
| cg03217795 |  | cg07555797 |  | cg03001333 |  | cg12718519 |  | cg22712840 |  |
| cg03306374 |  | cg13425637 |  | cg03580279 |  | cg12764620 |  | cg22865720 |  |
| cg04279973 |  | cg18729886 |  | cg03599988 |  | cg13072890 |  | cg22876683 |  |
| cg08406370 |  | cg20457147 |  | cg03732007 |  | cg13077150 |  | cg23803603 |  |
| cg24250393 |  | cg02973263 | PRKCI | cg03777518 |  | cg13387826 |  | cg23820589 |  |
| cg02121118 | PRKCD | cg06048350 |  | cg03959306 |  | cg13480549 |  | cg23844174 |  |
| cg02559423 |  | cg10800003 |  | cg04023573 |  | cg13591369 |  | cg24042578 |  |
| cg02630105 |  | cg12670756 |  | cg04315214 |  | cg13918804 |  | cg24138964 |  |
| cg07687398 |  | cg20695805 |  | cg04349178 |  | cg14171824 |  | cg24557048 |  |
| cg08170911 |  | cg24295479 |  | cg04520340 |  | cg14417954 |  | cg24776480 |  |
| cg10460643 |  | cg00613753 | PRKCQ | cg04771433 |  | cg14418633 |  | cg25139718 |  |
| cg13469346 |  | cg01389728 |  | cg05753589 |  | cg15481172 |  | cg25304754 |  |
| cg13889007 |  | cg05096182 |  | cg06157219 |  | cg15550572 |  | cg25319188 |  |
| cg13908523 |  | cg05456956 |  | cg06176608 |  | cg16048006 |  | cg25407448 |  |
| cg14727059 |  | cg09877009 |  | cg06204101 |  | cg16059943 |  | cg25466090 |  |
| cg20886263 |  | cg11638200 |  | cg06315149 |  | cg16599817 |  | cg25845463 |  |
| cg21903826 |  | cg12245996 |  | cg06717814 |  | cg16653138 |  | cg25976434 |  |
| cg06824134 | PRKCE | cg19910780 |  | cg06757383 |  | cg17023856 |  | cg26335530 |  |
| cg11476211 |  | cg23434428 |  | cg06791083 |  | cg17426766 |  | cg26561349 |  |
| cg11662638 |  | cg24107750 |  | cg06821641 |  | cg17495046 |  | cg26610624 |  |
| cg13547577 |  | cg24895834 |  | cg06888836 |  | cg17748900 |  | cg27587033 |  |
| cg18204079 |  | cg25741578 |  | cg07375700 |  | cg18188739 |  |  |  |
| cg19538885 |  | cg26617886 |  | cg07836663 |  | cg18269141 |  |  |  |
| cg21701125 |  | cg00171113 | PRKCZ | cg08106205 |  | cg18803110 |  |  |  |
| cg23861889 |  | cg00312809 |  | cg08106279 |  | cg18888671 |  |  |  |
| cg03657031 | PRKCG | cg00359465 |  | cg08651178 |  | cg19257562 |  |  |  |
| cg04518808 |  | cg00409816 |  | cg08732456 |  | cg19646139 |  |  |  |
| cg06697294 |  | cg00443516 |  | cg09322616 |  | cg19759051 |  |  |  |
| cg11662224 |  | cg00866690 |  | cg09560161 |  | cg19803229 |  |  |  |
| cg21950287 |  | cg00981070 |  | cg11227141 |  | cg20153240 |  |  |  |

**Table S3 Univariate and Multivariate analysis**

|  | | **OS** | | |  |  | | **PFI** | | |
| --- | --- | --- | --- | --- | --- | --- | --- | --- | --- | --- |
|  |  | **Univariate** | **Multivariate (Clinical Features)** | **Multivariate (Clinical + Histological Features)** |  |  |  | **Univariate** | **Multivariate (Clinical Features)** | **Multivariate (Clinical + Histological Features)** |
| **Cancer Type** | **Gene** | **HR (95% CI for HR) p.value** | | |  | **Cancer Type** | **Gene** | **HR (95% CI for HR) p.value** | | |
| **ACC** | **PRKCA** | 0.69 (0.58-0.81)0 | 0.64(0.53-0.79)0 |  |  | **ACC** | **PRKCA** | 0.71 (0.61-0.81)0 | 0.69(0.58-0.81)0 | ----- |
| **HNSC** | **PRKCA** | 1.2 (1.1-1.4)0 | 1.21(1.06-1.38)0 | **1.21(1.04-1.4)0.01** |  | **CESC** | **PRKCA** | 1.3 (1.1-1.5)0.01 | ----- | ----- |
| **LGG** | **PRKCA** | 0.49 (0.38-0.63)0 | 0.5(0.38-0.65)0 | **0.62(0.48-0.81)0** |  | **KIRP** | **PRKCA** | 0.61 (0.42-0.89)0 | 0.63(0.43-0.91)0.01 | ----- |
| **MESO** | **PRKCA** | 1.4 (1.1-1.8)0.01 | 1.42(0.70-1.08)0.01 | **1.53(1.14-2.04)0** |  | **LGG** | **PRKCA** | 0.51 (0.41-0.64)0 | 0.53(0.42-0.67)0 | **0.6(0.48-0.75)0** |
| **THCA** | **PRKCA** | 1.7 (1.2-2.6)0 | ----- | ----- |  | **LIHC** | **PRKCA** | 1.3 (1.1-1.6)0 | 1.27(1.05-1.54)0.01 |  |
| **UVM** | **PRKCA** | 1.8 (1.2-2.6)0 | 1.68(1.17-2.43)0 | ----- |  | **SKCM** | **PRKCA** | 0.91 (0.84-0.99)0.02 | 0.91(0.84-0.98)0.01 | **0.88(0.84-0.98)0** |
| **ACC** | **PRKCB** | 0.81 (0.67-0.99)0.03 | ----- | ----- |  | **UVM** | **PRKCA** | 2.2 (1.5-3.1)0 | 2.21(1.54-3.17)0 | **1.84(1.25-2.72)0** |
| **BRCA** | **PRKCB** | 0.9 (0.82-0.98)0.01 | 0.9(0.83-0.99)0.03 | ----- |  | **ACC** | **PRKCB** | 0.78 (0.66-0.91)0 | 0.79(0.67-0.93)0 | ----- |
| **CESC** | **PRKCB** | 0.78 (0.67-0.9)0 | 0.78(0.67-0.91)0 | **0.81(0.67-0.97)0.02** |  | **BRCA** | **PRKCB** | 0.88 (0.8-0.96)0 | 0.87(0.8-0.95)0 | ----- |
| **HNSC** | **PRKCB** | 0.87 (0.8-0.95)0 | 0.86(0.79-0.94)0 | **0.89(0.82-0.98)0.02** |  | **CESC** | **PRKCB** | 0.78 (0.67-0.9)0 | 0.79(0.67-0.92)0 | ----- |
| **LGG** | **PRKCB** | 0.76 (0.66-0.86)0 | 0.75(0.66-0.86)0 | **0.79(0.70-0.9)0** |  | **CHOL** | **PRKCB** | 0.79 (0.63-0.99)0.04 | 0.72(0.55-0.94)0.01 | ----- |
| **LUAD** | **PRKCB** | 0.83 (0.74-0.93)0 | 0.79(0.70-0.89)0 | **0.79(0.69-0.9)0** |  | **HNSC** | **PRKCB** | 0.87 (0.8-0.96)0 | 0.87(0.79-0.95)0 | **0.87(0.79-0.96)0.01** |
| **PCPG** | **PRKCB** | 2 (1.3-2.9)0 | 2.08(1.38-3.13)0 | **1.56(1.07-2.28)0.02** |  | **LGG** | **PRKCB** | 0.83 (0.75-0.92)0 | 0.81(0.73-0.9)0 | **0.85(0.77-0.94)0** |
| **SARC** | **PRKCB** | 0.87 (0.79-0.97)0 | 0.84(0.75-0.94)0 | **0.88(0.79-0.99)0.04** |  | **LIHC** | **PRKCB** | 0.86 (0.77-0.96)0 | 0.84(0.75-0.94)0 | **0.82(0.73-0.91)0** |
| **SKCM** | **PRKCB** | 0.92 (0.87-0.98)0.01 | 0.92(0.86-0.98)0.01 | ----- |  | **LUAD** | **PRKCB** | 0.88 (0.79-0.98)0.02 | 0.87(0.78-0.98)0.02 | ----- |
| **ACC** | **PRKCD** | 0.56 (0.37-0.84)0 | 0.5(0.32-0.78)0 | ----- |  | **PCPG** | **PRKCB** | 1.6 (1.3-2)0 | 1.62(1.32-1.99)0 | **1.37(1.11-1.68)0** |
| **BLCA** | **PRKCD** | 0.78 (0.65-0.93)0 | 0.8(0.67-0.97)0.02 | ----- |  | **PRAD** | **PRKCB** | 0.85 (0.73-0.98)0.02 | ----- | ----- |
| **LAML** | **PRKCD** | 1.3 (1-1.5)0.02 | 1.23(1.01-1.51)0.03 | ----- |  | **ACC** | **PRKCD** | 0.67 (0.48-0.95)0.02 | 0.67(0.48-0.94)0.02 | **0.65(0.43-0.99)0.04** |
| **LGG** | **PRKCD** | 1.2 (1-1.4)0.02 | 1.38(1.16-1.65)0 | **1.3(1.09-1.56)0** |  | **BLCA** | **PRKCD** | 0.72 (0.6-0.86)0 | 0.73(0.6-0.88)0 | **0.79(0.65-0.96)0.02** |
| **LIHC** | **PRKCD** | 1.4 (1.2-1.7)0 | 1.49(1.22-1.83)0 | **1.38(1.10-1.73)0** |  | **GBM** | **PRKCD** | 1.3 (1-1.6)0.04 | 1.34(1.05-1.72)0.01 | ----- |
| **LUAD** | **PRKCD** | 0.59 (0.47-0.73)0 | 0.56(0.45-0.7)0 | **0.58(0.46-0.74)0** |  | **LGG** | **PRKCD** | 1.3 (1.1-1.4)0 | 1.33(1.15-1.52)0 | **1.27(1.1-1.47)0** |
| **THCA** | **PRKCD** | 0.17 (0.043-0.71)0.01 | ----- | 2.11(1.05-4.25)0.03 |  | **LUAD** | **PRKCD** | 0.71 (0.56-0.88)0 | 0.7(0.55-0.89)0 | ----- |
| **UVM** | **PRKCD** | 0.31 (0.17-0.55)0 | 0.3(0.16-0.55)0 | ----- |  | **PRAD** | **PRKCD** | 1.7 (1.1-2.6)0.02 | ----- | ----- |
| **KIRC** | **PRKCE** | 0.6 (0.51-0.71)0 | 0.59(0.50-0.69)0 | **0.67(0.56-0.81)0** |  | **READ** | **PRKCD** | 3.5 (1.2-10)0.02 | 3.54(1.22-10.2)0.01 | **81.2(7.65-862)0** |
| **KIRP** | **PRKCE** | 1.4 (1-2)0.04 | ----- | **1.54(1.03-2.31)0.03** |  | **UVM** | **PRKCD** | 0.33 (0.2-0.54)0 | 0.32(0.19-0.55)0 | **0.45(0.25-0.82)0** |
| **LGG** | **PRKCE** | 0.58 (0.47-0.71)0 | 0.6(0.49-0.74)0 | **0.69(0.57-0.84)0** |  | **KIRC** | **PRKCE** | 0.59 (0.5-0.69)0 | 0.59(0.5-0.7)0 | ----- |
| **LUAD** | **PRKCE** | 0.73 (0.6-0.88)0 | 0.71(0.58-0.86)0 | **0.64(0.52-0.78)0** |  | **LGG** | **PRKCE** | 0.66 (0.57-0.77)0 | 0.67(0.57-0.78)0 | **0.69(0.59-0.81)0** |
| **KIRC** | **PRKCG** | 1.3 (1.2-1.4)0 | 1.38(1.25-1.52)0 | **1.21(1.08-1.37)0** |  | **PAAD** | **PRKCE** | 0.64 (0.49-0.83)0 | 0.59(0.43-0.81)0 | ----- |
| **LGG** | **PRKCG** | 0.92 (0.86-0.99)0.02 | 0.9(0.84-0.97)0 | ----- |  | **PCPG** | **PRKCE** | 0.53 (0.35-0.81)0 | 0.52(0.33-0.8)0 | ----- |
| **PAAD** | **PRKCG** | 0.89 (0.81-0.97)0 | 0.89(0.82-0.98)0.02 | ----- |  | **STAD** | **PRKCE** | 1.3 (1-1.7)0.02 | 1.38(1.05-1.82)0.01 |  |
| **PCPG** | **PRKCG** | 1.4 (1.1-1.7)0 | 1.56(1.21-2)0 | **1.29(1.00-1.66)0.04** |  | **UVM** | **PRKCE** | 2.9 (1.4-5.8)0 | 2.76(1.37-5.58)0 | **3.7(1.53-8.99)0** |
| **SKCM** | **PRKCG** | 0.73 (0.6-0.88)0 | 0.75(0.61-0.91)0 | ----- |  | **ACC** | **PRKCG** | 1.4 (1.1-1.6)0 | 1.35(1.1-1.66)0 | **1.3(1.01-1.68)0.03** |
| **THYM** | **PRKCG** | 0.6 (0.43-0.83)0 | 0.62(0.44-0.89)0 | ----- |  | **KIRC** | **PRKCG** | 1.2 (1.1-1.4)0 | 1.27(1.13-1.42)0 | ----- |
| **ACC** | **PRKCH** | 0.73 (0.54-0.99)0.04 | ----- |  |  | **LGG** | **PRKCG** | 0.94 (0.89-1)0.04 | 0.93(0.88-0.99)0.02 | ----- |
| **CESC** | **PRKCH** | 0.71 (0.55-0.93)0.01 | 0.72(0.55-0.95)0.02 | **0.74(0.56-0.98)0.03** |  | **PCPG** | **PRKCG** | 1.6 (1.4-1.9)0 | 1.6(1.37-1.86)0 | **1.36(1.14-1.63)0** |
| **HNSC** | **PRKCH** | 0.75 (0.6-0.94)0.01 | ----- | 0.78(0.62-0.99)0.04 |  | **SKCM** | **PRKCG** | 0.86 (0.74-0.99)0.03 | ----- | ----- |
| **KIRC** | **PRKCH** | 0.72 (0.61-0.85)0 | 0.7(0.58-0.83)0 | **0.77(0.64-0.93)0** |  | **THYM** | **PRKCG** | 0.86 (0.74-0.99)0.04 | 0.8(0.68-0.94)0.01 | ----- |
| **LGG** | **PRKCH** | 1.4 (1.1-1.8)0 | 1.41(1.10-1.81)0 | ----- |  | **ACC** | **PRKCH** | 0.7 (0.55-0.88)0 | 0.72(0.56-0.93)0.01 | ----- |
| **LIHC** | **PRKCH** | 0.75 (0.63-0.9)0 | 0.7(0.58-0.85)0 | **0.760.626-0.93)0** |  | **BRCA** | **PRKCH** | 0.76 (0.62-0.92)0 | 0.76(0.62-0.93)0 | **0.81(0.66-0.99)0.04** |
| **LUAD** | **PRKCH** | 0.75 (0.59-0.95)0.01 | 0.74(0.58-0.95)0.01 | **0.71(0.54-0.93)0.01** |  | **CESC** | **PRKCH** | 0.66 (0.51-0.85)0 | 0.66(0.5-0.85)0 | **0.69(0.52-0.91)0** |
| **SKCM** | **PRKCH** | 0.86 (0.77-0.97)0.01 | 0.88(0.78-0.99)0.03 | ----- |  | **CHOL** | **PRKCH** | 0.56 (0.35-0.89)0.01 | 0.51(0.31-0.85)0 | ----- |
| **STAD** | **PRKCH** | 1.2 (1-1.5)0.04 | 1.29(0.78-0.99)0.01 | ----- |  | **HNSC** | **PRKCH** | 0.72 (0.57-0.9)0 | 0.72(0.57-0.91)0 | **0.72(0.57-0.92)0** |
| **UVM** | **PRKCH** | 0.59 (0.37-0.93)0.02 | 0.53(0.31-0.91)0.02 | ----- |  | **KIRC** | **PRKCH** | 0.74 (0.62-0.88)0 | 0.72(0.6-0.86)0 | ----- |
| **ACC** | **PRKCI** | 2.3 (1.2-4.4)0.01 | 2.19(1.12-4.29)0.02 | ----- |  | **LGG** | **PRKCH** | 1.2 (1-1.5)0.04 | 1.24(1.01-1.52)0.03 | ----- |
| **KICH** | **PRKCI** | 2.1 (1.1-3.9)0.01 | ----- | ----- |  | **LIHC** | **PRKCH** | 0.76 (0.65-0.9)0 | 0.71(0.6-0.84)0 | **0.68(0.56-0.82)0** |
| **LAML** | **PRKCI** | 0.6 (0.4-0.91)0.01 | ----- | ----- |  | **STAD** | **PRKCH** | 1.3 (1.1-1.6)0 | 1.38(1.1-1.71)0 | ----- |
| **LGG** | **PRKCI** | 1.8 (1-3.1)0.03 | 1.73(1.00-3)0.04 | ----- |  | **THCA** | **PRKCH** | 0.49 (0.33-0.74)0 | 0.53(0.34-0.8)0 | ----- |
| **LIHC** | **PRKCI** | 1.4 (1.1-1.8)0.01 | 1.45(1.10-1.91)0 | ----- |  | **UVM** | **PRKCH** | 0.56 (0.33-0.96)0.03 | ----- | ----- |
| **PAAD** | **PRKCI** | 1.7 (1.3-2.2)0 | 1.73(1.32-2.27)0 | **1.9(1.19-3.04)0** |  | **KICH** | **PRKCI** | 2.2 (1.2-4.3)0.01 | 2(1.02-3.9)0.04 | **2.61(1.04-6.55)0.03** |
| **SARC** | **PRKCI** | 1.7 (1.2-2.4)0 | 1.68(1.14-2.49)0 | ----- |  | **LIHC** | **PRKCI** | 1.3 (1-1.6)0.02 | 1.27(0.76-0.93)0.03 | ----- |
| **ACC** | **PRKCQ** | 1.2 (1-1.4)0.04 | ----- | ----- |  | **PAAD** | **PRKCI** | 1.7 (1.3-2.1)0 | 1.66(1.3-2.11)0 | ----- |
| **BRCA** | **PRKCQ** | 0.91 (0.83-0.99)0.03 | ----- | ----- |  | **THYM** | **PRKCI** | 0.51 (0.28-0.96)0.03 | 0.5(0.25-0.99)0.04 | ----- |
| **KICH** | **PRKCQ** | 0.61 (0.41-0.91)0.01 | 0.52(0.31-0.85)0 | **0.52(0.32-0.83)0** |  | **UCEC** | **PRKCI** | 1.4 (1.1-1.9)0.02 | 1.35(0.25-0.99)0.04 | ----- |
| **LGG** | **PRKCQ** | 0.81 (0.68-0.96)0.01 | 0.81(0.68-0.96)0.02 | ----- |  | **ACC** | **PRKCQ** | 1.2 (1-1.3)0.04 | ----- | ----- |
| **LIHC** | **PRKCQ** | 0.86 (0.76-0.96)0 | 0.84(0.74-0.95)0 | ----- |  | **BRCA** | **PRKCQ** | 0.89 (0.81-0.98)0.01 | 0.88(0.81-0.97)0.01 | ----- |
| **SKCM** | **PRKCQ** | 0.87 (0.82-0.93)0 | 0.88(0.82-0.94)0 | ----- |  | **LGG** | **PRKCQ** | 0.84 (0.72-0.97)0.01 | 0.84(0.73-0.97)0.02 | ----- |
| **THYM** | **PRKCQ** | 0.6 (0.4-0.9)0.01 | ----- | ----- |  | **LIHC** | **PRKCQ** | 0.85 (0.77-0.94)0 | 0.84(0.76-0.93)0 | **0.86(0.78-0.96)0** |
| **UCEC** | **PRKCQ** | 0.84 (0.72-0.98)0.02 | 0.85(0.73-0.98)0.03 | ----- |  | **PCPG** | **PRKCQ** | 1.3 (1.1-1.6)0 | 1.3(1.07-1.59)0 | ----- |
| **BRCA** | **PRKCZ** | 0.73 (0.59-0.9)0 | 0.73(0.59-0.9)0 | **0.73(0.58-0.92)0** |  | **SKCM** | **PRKCQ** | 0.94 (0.89-0.99)0.01 | 0.94(0.89-0.99)0.03 | ----- |
| **KICH** | **PRKCZ** | 0.31 (0.12-0.79)0.01 | ----- | ----- |  | **BRCA** | **PRKCZ** | 0.77 (0.62-0.95)0.01 | 0.74(0.6-0.92)0 | ----- |
| **KIRC** | **PRKCZ** | 0.72 (0.6-0.85)0 | 0.71(0.59-0.85)0 | ----- |  | **KIRC** | **PRKCZ** | 0.71 (0.61-0.84)0 | 0.73(0.61-0.86)0 | ----- |
| **KIRP** | **PRKCZ** | 0.47 (0.3-0.76)0 | 0.44(0.27-0.74)0 | ----- |  | **KIRP** | **PRKCZ** | 0.53 (0.35-0.79)0 | 0.54(0.43-0.91)0 | ----- |
| **LGG** | **PRKCZ** | 0.69 (0.59-0.81)0 | 0.73(0.62-0.86)0 | **0.82(0.70-0.96)0.01** |  | **LGG** | **PRKCZ** | 0.8 (0.7-0.91)0 | 0.82(0.72-0.94)0 | **0.86(0.76-0.99)0.03** |
| **LUAD** | **PRKCZ** | 0.81 (0.69-0.93)0 | 0.8(0.69-0.94)0 | **0.76(0.66-0.88)0** |  | **LUSC** | **PRKCZ** | 1.3 (1-1.6)0.04 | ----- | 1.38(1.07-1.78)0.01 |
| **MESO** | **PRKCZ** | 0.65 (0.51-0.82)0 | 0.65(0.51-0.83)0 | **0.61(0.46-0.81)0** |  | **UVM** | **PRKCZ** | 0.67 (0.48-0.92)0.01 | 0.69(0.49-0.96)0.02 |  |

**Supplementary figure legends**

**Figure S1:** Heatmap of the significant PKC genes expression differences in cancers compared to normal tissues in different cancer types, the red color indicates elevated expression in cancer, the blue color indicates low expression in cancer. *: p-value < 0.05; **: p-value < 0.01; ***: p-value < 0.001; ****: p-value < 0.0001

**Figure S2:** Heatmap of the significant PKC genes promotors methylation level differences in cancers compared to normal tissues in different cancer types, the red color indicates elevated methylation level in cancer, the blue color indicates low methylation level in cancer. *: p-value < 0.05; **: p-value < 0.01; ***: p-value < 0.001; ****: p-value < 0.0001.

**Figure S3:** Significant correlation coefficients between PKC genes expressions and ESTIMATE immune scores of different cancer types. The color represent the correlation coefficients.*: p value < 0.05; **: p value < 0.01; ***: p value < 0.001.


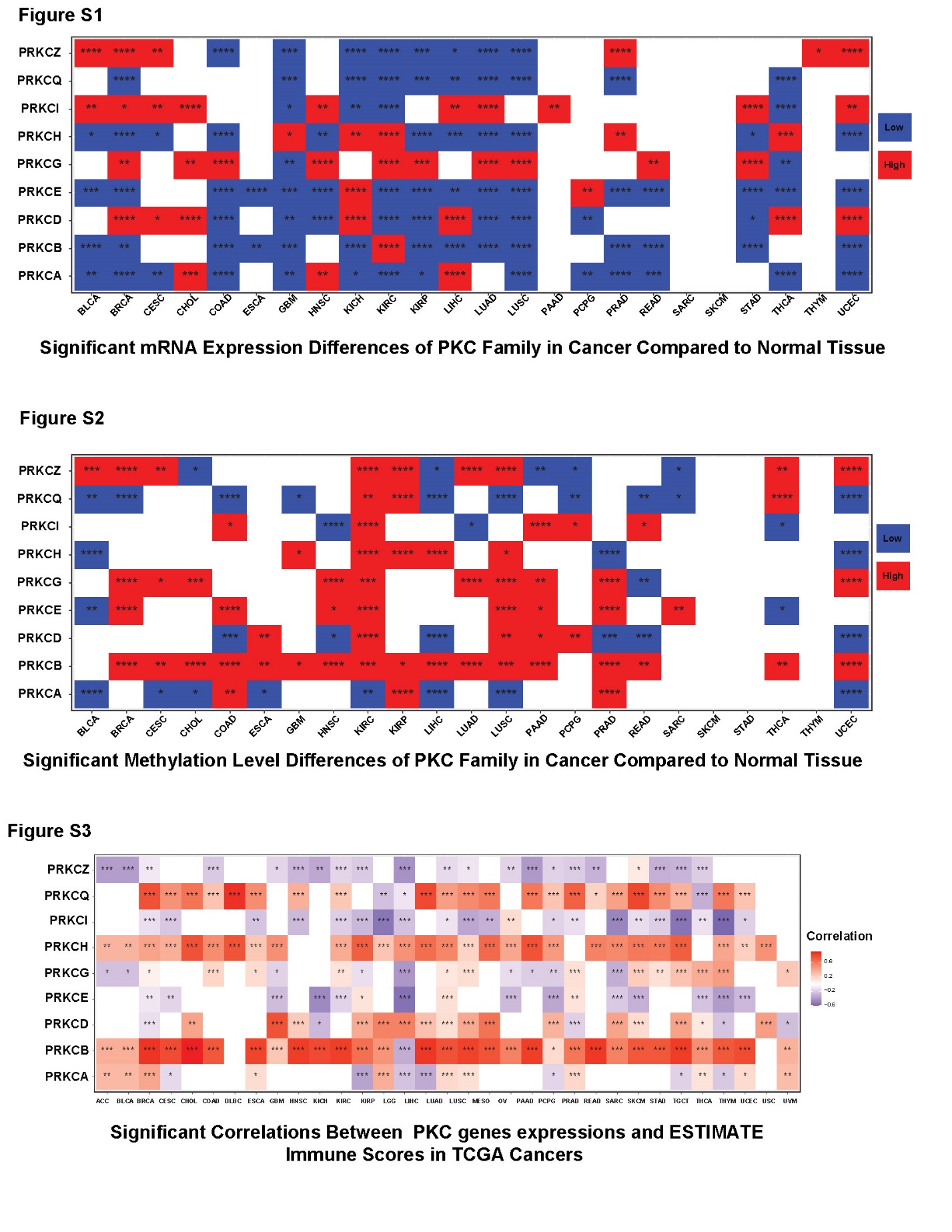

Supplement: Supplementary file 1 [file DataSheet1.docx]
